# Delphinidin-3-O-glucoside attenuates neonatal hypoxic-ischemic encephalopathy in a neonatal mouse model by reprogramming microglial polarization

**DOI:** 10.3389/fphar.2026.1797078

**Published:** 2026-05-04

**Authors:** Jiacan Xu, Pengyu Hao, Jie Chen, Diqi Mai, Ying Wang, Ran Chen, Jiani Yang, Renjie Li, Xiaoyu Xing, Ziqiao Yan, Jianing Cao, Yilin Sun, Bodong Wang

**Affiliations:** 1 Department of Neurosurgery, the 960th Hospital of PLA (General Hospital of Jinan Military Command), Jinan, Shandong, China; 2 Department of Histology and Embryology, School of Basic Medical Science, Qilu College of Medicine, Shandong University, Jinan, Shandong, China; 3 School of Clinical Medicine, Shandong Second Medical University, Weifang, Shandong, China; 4 School of Clinical Medicine, Shandong First Medical University, Jinan, Shandong, China

**Keywords:** delphinidin-3-O-glucoside, hypoxic-ischemic encephalopathy, microglial polarization, neuroinflammation, neuroprotection, oxidative stress

## Abstract

**Background:**

Cerebral ischemic injury includes ischemic stroke in adults and hypoxic-ischemic encephalopathy (HIE) in newborns. Delphinidin-3-O-glucoside (Dp3G) is a bioactive anthocyanin abundant in vibrantly colored plant-based foods and known for its potent antioxidant properties. Previous studies have shown that Dp3G suppresses oxidative stress and inflammation in atherosclerosis, protects against myocardial ischemic injury, and sensitizes glioblastoma to temozolomide by reducing drug resistance. However, its role and mechanisms in hypoxic-ischemic brain injury remain unclear.

**Methods:**

This study investigated the neuroprotective effects and mechanisms of Dp3G using *in vitro* and *in vivo* models. An oxygen–glucose deprivation (OGD) model in microglia was used for in vitro assessment. In vivo, a neonatal KM mouse model of HIE was employed. Techniques included transcriptomic analysis and molecular docking to elucidate the underlying mechanisms.

**Results:**

In a microglial oxygen–glucose deprivation (OGD) model, Dp3G reduced apoptosis, inflammatory responses, and oxidative stress. Transcriptomic analysis suggested that Dp3G promotes metabolic reprogramming and inhibits NF-κB signaling via NLRC3, shifting microglial polarization toward an anti-inflammatory phenotype. Molecular docking indicated a potential interaction between Dp3G and NLRC3. In a neonatal KM mouse model of HIE, Dp3G treatment reduced neuronal damage and cerebral infarction and restored regional cerebral blood flow, with mechanistic evidence supporting microglial polarization regulation.

**Conclusion:**

Dp3G exerts significant neuroprotective effects in models of hypoxic-ischemic brain injury. Its mechanism involves the modulation of microglial polarization, and interaction with NLRC3 and subsequent inhibition of NF-κB signaling. These findings identify Dp3G as a promising neuroprotective candidate in preclinical models of hypoxic-ischemic brain injury.

## Graphical Abstract

**Figure:**
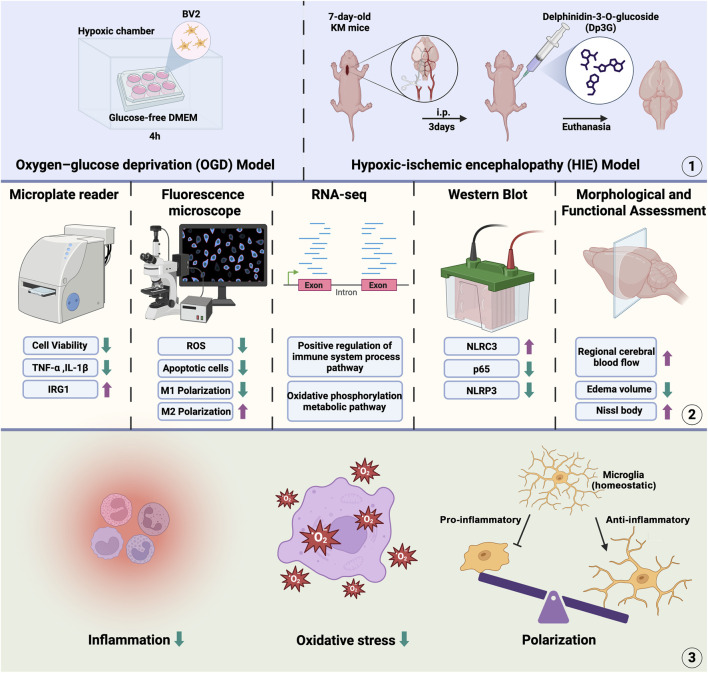

## 1 Introduction

Neonatal hypoxic-ischemic encephalopathy (HIE) is a severe neurological disorder that primarily affects full-term and near-term newborns and commonly results from perinatal hypoxic or ischemic events (Serrenho et al., 2021; Bäcke et al., 2022; Bruschettini et al., 2020). Epidemiological studies indicate that the incidence of HIE ranges from 1 to 8 cases per 1,000 live births in developed countries, increasing to approximately 26 per 1,000 in developing regions (Brégère et al., 2021; Maiza et al., 2024; Paz and González-Candia, 2023). As a leading cause of neonatal mortality, HIE leaves up to 60% of survivors with long-term neurodevelopmental impairments, including cerebral palsy, epilepsy, intellectual disability, learning difficulties, and speech or motor disorders (Ambalavanan et al., 2021; Tran et al., 2021; Babbo et al., 2024; He et al., 2024).

Microglia, the primary immune cells of the central nervous system, play a pivotal role in post-hypoxic-ischemic inflammatory responses and tissue repair (Nunes et al., 2025). These cells exhibit dual activation states. Classical polarization toward the pro-inflammatory phenotype in response to hypoxia-ischemia leads to the release of pro-inflammatory cytokines, reactive oxygen species (ROS), and reactive nitrogen species (RNS), thereby exacerbating neuronal damage. In contrast, alternative activation promotes the anti-inflammatory phenotype, which secretes neuroprotective factors and facilitates tissue regeneration and repair (Wang et al., 2025; Lemanski et al., 2024; Chen et al., 2022; Rayasam et al., 2021). Under pathological conditions, an imbalance between pro-inflammatory pro-inflammatory and anti-inflammatory anti-inflammatory polarization drives uncontrolled neuroinflammation, highlighting microglial polarization as a promising therapeutic target for hypoxic-ischemic brain injury.

**FIGURE 1:**
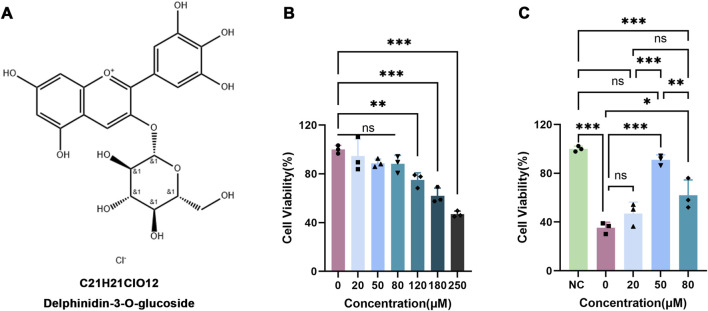
Effects of Dp3G on BV2 cell viability. **(A)** Structural formula and molecular formula of Dp3G; **(B)** CCK-8 assay measuring BV2 cell viability after 24 h treatment with different concentrations of Dp3G; **(C)** CCK-8 assay measuring BV2 cell viability after OGD treatment with different concentrations of Dp3G. Data represent the mean ± standard error of the mean (SEM) from n = 3 per condition. Statistical significance was assessed using one-way analysis of variance (ANOVA). *p < 0.05, **p < 0.01, ***p < 0.001.

Anthocyanins, a class of water-soluble flavonoids widely present in plants, have attracted increasing attention because of their multi-target bioactivities, favorable safety profile, and potent antioxidant and anti-inflammatory properties (Liobikas et al., 2016; Wei et al., 2025). In the context of neuroprotection, anthocyanins prevent neuronal death by scavenging mitochondrial ROS (Brown and Borutaite, 2012). For example, cyanidin-3-glucoside (Cy3glc), one of the active anthocyanin monomers, reduces cerebral infarct volume and improves neurological function in a mouse model of cerebral artery occlusion ischemia (Min et al., 2011). Furthermore, anthocyanins exert protective effects in a Parkinson’s disease model by ameliorating rotenone-induced dysfunction of mitochondrial Complex I (Strathearn et al., 2014; Brewer et al., 2010; Fuentealba et al., 2011). Notably, both Cy3glc and delphinidin-3-O-glucoside (Dp3G) can serve as electron acceptors for Complex I, promoting NADH oxidation and transferring electrons to cytochrome c, thereby sustaining mitochondrial electron transport chain function (Skemiene et al., 2015). Dp3G (Figure 1A) is one of the most active anthocyanin monomers (Ragab et al., 2022; Wu et al., 2024) and exhibits cytoprotective effects in various disease models. For example, Dp3G alleviates oxidative stress and inflammation in high-fat diet-induced atherosclerosis (Sun et al., 2022), restores mitochondrial energy metabolism in myocardial ischemia (Skemiene et al., 2015), and protects vascular endothelial cells in atherosclerosis via the SGLT1-ROS-mitochondrial pathway (Jin et al., 2013). However, although prior evidence suggests that Dp3G mitigates inflammation-induced stress signaling in microglia (Carey et al., 2013), it remains unclear whether Dp3G crosses the blood–brain barrier (BBB) to exert its anti-inflammatory and antioxidant neuroprotective effects *in vivo*, and whether it alleviates HIE by reprogramming microglial polarization. The underlying molecular targets and mechanisms also require further elucidation.

Based on the above evidence, we propose the following hypothesis: Dp3G can cross the BBB and, through its anti-inflammatory and antioxidant effects, reprogram microglial status, shifting microglia from a pro-inflammatory phenotype to an anti-inflammatory phenotype, thereby ultimately alleviating neuronal injury in a neonatal mouse model of HIE. To test this hypothesis, the present study systematically investigated the neuroprotective effects and mechanisms of Dp3G in hypoxic-ischemic brain injury., *In vitro* experiments demonstrated that Dp3G reprograms BV2 cell polarization following oxygen–glucose deprivation (OGD), promoting a shift from the pro-inflammatory to the anti-inflammatory phenotype. This shift induces anti-inflammatory and antioxidant effects, thereby protecting neurons from injury. RNA sequencing analysis further revealed that the therapeutic effects of Dp3G were enriched in pathways related to oxidative phosphorylation and immune system regulation. In addition, molecular docking predicted potential protein targets of Dp3G, providing mechanistic clues for subsequent functional validation. Finally, in a neonatal mouse model of hypoxic-ischemic brain injury, Dp3G treatment mitigated cerebral edema, restored cerebral blood flow, reduced cell death, and shifted microglial polarization from the detrimental pro-inflammatory phenotype toward the beneficial anti-inflammatory phenotype.

In summary, this study is the first to demonstrate the protective effects of Dp3G against hypoxic-ischemic brain injury. The findings suggest that Dp3G may, on one hand, reprogram microglial metabolism by promoting ACOD1 (IRG1) expression and increasing the production of the anti-inflammatory metabolite itaconate, and on the other hand, inhibit the NF-κB pathway by upregulating NLRC3. These two mechanisms act synergistically to promote microglial polarization toward an anti-inflammatory phenotype, thereby indirectly protecting neurons. Furthermore, molecular docking identified NLRC3 as a potential activation target of Dp3G. This work provides preclinical evidence and a theoretical basis for developing Dp3G as a natural product-derived neuroprotective agent.

## 2 Materials and methods

### 2.1 Cell culture

The BV2 microglial cells (provided by the National Collection of Authenticated Cell Cultures, China) and HT22 neuronal cells (provided by the National Collection of Authenticated Cell Cultures, China) were cultured in high-glucose DMEM (Gibco, Carlsbad, CA, United States) supplemented with 10% FBS (Gibco, Carlsbad, CA, United States) and 1% penicillin (100 U/mL)/streptomycin (100 μg/mL) (Gibco, Carlsbad, CA, United States) under 5% CO_2_ at 37 °C. BV2 is an immortalized murine microglial cell line, and HT22 is an immortalized murine hippocampal neuronal cell line. Both are widely used as *in vitro* models for neurobiological studies.

### 2.2 Oxygen–glucose deprivation (OGD) model establishment

BV2 cells were seeded in 6-well plates. When cell density reached 60%–70%, cells were washed with PBS and incubated in glucose-free DMEM (Gibco, Carlsbad, CA, United States). Plates were then transferred to a hypoxic chamber and maintained under hypoxic conditions for 4 h. After treatment, cells were collected for subsequent experiments.

### 2.3 Cell viability assay

Cell viability of BV2 cells under OGD conditions with Dp3G (MCE, Shanghai, China) treatment was assessed using the Cell Counting Kit-8 (CCK-8) assay. BV2 cells in the logarithmic growth phase were seeded in 96-well plates at a density of 5 × 10^4^ cells per well. After treatment with various concentrations of Dp3G (20, 50, 80, 120, 180, and 250 μM) dissolved in DMSO and OGD exposure for 4 h, 10 μL of CCK-8 solution (Beyotime Biotechnology, Shanghai, China) was added to each well and incubated for 1 h. Absorbance at 450 nm was measured using a microplate reader (Allsheng, Hangzhou, China) for subsequent statistical analysis.

### 2.4 Measurement of intracellular ROS levels

Intracellular ROS levels in BV2 cells were assessed using the fluorescent probe DCFH-DA (Beyotime Biotechnology, Shanghai, China). Cells were seeded in 6-well plates and allowed to grow to 60%–70% confluence. The culture medium was removed, and cells were incubated with 1 mL of DCFH-DA working solution (diluted in PBS to a final concentration of 10 μM) in the dark at 37 °C for 20 min. After incubation, cells were washed three times with ice-cold PBS to remove unincorporated probe. For nuclear staining, cells were counterstained with Hoechst solution for 15 min, followed by three washes with PBS. Fluorescence images were acquired using a laser confocal scanning microscope (OLYMPUS CKX53). DCFH-DA fluorescence was detected at excitation and emission wavelengths of 488 nm and 525 nm, respectively.

### 2.5 JC-1 staining

Mitochondrial membrane potential (Ψm) was assessed using a JC-1 assay kit (Beyotime Biotechnology, Shanghai, China). HT22 cells were seeded in 6-well plates and allowed to grow to 60%–70% confluence. The culture medium was removed and add 1 mL of cell culture medium (which may contain serum and phenol red). Add 1 mL of JC-1 staining solution and mix thoroughly. Incubate in a cell culture incubator at 37 °C for 20 min. During incubation, prepare an appropriate amount of JC-1 staining buffer (1X) by adding 4 mL of distilled water for every 1 mL of JC-1 staining buffer (5X), and place it on ice. After incubation at 37 °C, aspirate the supernatant and wash twice with JC-1 staining buffer (1X). Add 2 mL of cell culture medium. Observe under a laser confocal microscope (OLYMPUS CKX53). When detecting JC-1 monomers, set the excitation wavelength to 490 nm and the emission wavelength to 530 nm; when detecting JC-1 polymers, set the excitation wavelength to 525 nm and the emission wavelength to 590 nm.

### 2.6 RNA extraction and qRT-PCR analysis

Total RNA was extracted from cultured BV2 cells using the TRIzol reagent method. Approximately 5 × 10^6^ cells were collected and lysed in 1 mL of TRIzol reagent (Thermo Fisher Scientific, MA, United States). Following homogenization, isopropanol was added to precipitate RNA, and samples were centrifuged at 4 °C. The resulting RNA pellet was washed with an equal volume of 75% ethanol, centrifuged again, and dissolved in 0.5% SDS.

Extracted RNA was reverse-transcribed into cDNA. Quantitative PCR was performed using a real-time PCR system with gene-specific primers for iNOS, CD206, ACOD1 and β-actin, together with SYBR Green master mix (SparkeJade, Shandong, China). mRNA expression levels of iNOS, CD206, ACOD1 and β-actin were normalized to an appropriate internal control and analyzed using the comparative Ct method. qRT-PCR primer sequences are shown in Supplementary Table S1.

### 2.7 ELISA

Concentrations of TNF-α, IL-1β, IL-10 and IRG1 in culture supernatants from BV2 cells following OGD exposure and Dp3G treatment were quantified using specific ELISA kits (Sangon Biotech, Shanghai, China) according to the manufacturer’s instructions. Collected supernatants were centrifuged at 120 *g* for 10 min to remove cellular debris. The resulting supernatants were analyzed using a double-antibody sandwich ELISA protocol to determine cytokine concentrations.

### 2.8 Bulk RNA-seq analysis

Transcriptomic analysis of BV2 cell samples from the OGD and Dp3G groups was performed by a commercial service provider using bulk RNA-seq. DEGs; |log_2_FC| > 1.5, *p* < 0.05) were subjected to functional annotation using GO and KEGG enrichment analyses.

### 2.9 Western blot analysis

Protein was extracted from BV2 cells. For cell samples, BV2 cells were collected after OGD treatment and lysed with 0.5 mL of pre-cooled lysis buffer per 5 × 10^6^ cells. Lysates were centrifuged at 16,000 rpm at 4 °C, and supernatants were collected for protein concentration determination. After normalization, samples were prepared by adding 2× Laemmli sample buffer and subjected to SDS-PAGE. Separated proteins were transferred to a PVDF membrane, which was blocked at room temperature for 2 h. Then membrane was then washed with Tris-Buffered Saline with Tween-20 (TBST). Incubate the membrane with NLRC3 (1:1000, Affinity, DF13411), NLRP3 (1:2000, Abclonal, A5652), and p65 (1:5000, Abclonal, A2547) at 4 °C overnight. The membrane was then washed with TBST, followed by incubation with HRP-conjugated secondary antibodies specific to the experimental species (1:10000, Proteintech, SA00001-2) at room temperature for 2 h, and finally washed with TBST. Signals were developed using an ECL kit (Bioprimacy, Wuhan, China) and detected with a chemiluminescence imaging system (Tanon-4800M).

### 2.10 Molecular docking and dynamics simulations

The Dp3G–NLRC3 interaction was modeled by molecular docking using AutoDock Vina. The resulting complex underwent a 100 ns all-atom MD simulation in an explicit solvent system using GROMACS after standard energy minimization and equilibration. Trajectory analysis included RMSD, RMSF, and hydrogen bond calculations.

### 2.11 HIE model establishment

A neonatal mouse model of HIE was established using the classical Rice–Vannucci procedure (Ditelberg et al., 1996). Briefly, postnatal day 7 KM mice were anesthetized with isoflurane (RWD, Shenzhen, China) and positioned supine on a sterile surgical platform. After skin disinfection with 75% ethanol, a midline cervical incision (approximately 5–10 mm) was made. The right anterior cervical muscles were carefully dissected using ophthalmic forceps to expose the carotid sheath. The right common carotid artery was gently isolated from the vagus nerve using a glass dissecting needle and permanently ligated with a 4-0 silk suture to ensure complete occlusion. The wound was sutured and disinfected, and the entire surgical procedure was completed within 10 min.

Following surgery, pups were placed on bedding with maternal scent for 30 min to recover before being returned to the dam for an additional hour. The mice were then placed in a 37 °C sealed hypoxia chamber infused with a gas mixture of 8% oxygen and 92% nitrogen for 50 min. Animals were randomly assigned to the Sham, HIE, and Dp3G groups using a random number table. After hypoxia induction, pups were administered Dp3G (20 mg/kg) by intraperitoneal (i.p.) injection once daily for three consecutive days, after which brains were harvested for subsequent analysis.

### 2.12 Measurement of rCBF

Regional cerebral blood flow (rCBF) was monitored using a laser speckle imaging system (MoorFLPI, Axminster, United Kingdom). The scalp of the pup was incised along the midline to fully expose the cranium and cerebrum, after which the camera was positioned perpendicularly 15 cm above the skull. Cerebral blood flow was recorded over a 60-s period in arbitrary PU after stabilization of perfusion signals.

### 2.13 TTC staining

To assess cerebral infarction, 2,3,5-triphenyltetrazolium chloride (TTC) staining was performed. Mice were euthanized at defined time points after HIE induction, and brains were rapidly removed. Five consecutive 1 mm-thick coronal sections were prepared, incubated in 2% TTC solution (Thermo Fisher Scientific, MA, United States) at 37 °C for 15 min, and fixed in 4% paraformaldehyde. Sections were photographed under standardized lighting conditions, and infarct volume was quantified using ImageJ software (version 1.54p).

### 2.14 Nissl staining

After fixation in 4% paraformaldehyde (Beyotime Biotechnology, Shanghai, China), whole brain tissues were paraffin-embedded and sectioned. After drying, sections were deparaffinized and dehydrated through a graded ethanol series. Sections were then rinsed with distilled water and immersed in Nissl staining solution (Solarbio, Beijing, China). After staining, sections were differentiated with 0.1% glacial acetic acid, with differentiation monitored under a microscope. The reaction was terminated by rinsing with water. Sections were then dried, cleared in xylene, mounted with neutral balsam, and examined under an optical microscope (OLYMPUS CKX53) to evaluate neuronal Nissl bodies.

### 2.15 TUNEL staining

Apoptotic cells were quantified using the TUNEL BrightRed Apoptosis Detection Kit (Vazyme, Nanjing, China). Brain tissue samples were fixed in 4% paraformaldehyde for 24 h, dehydrated in a gradient of ethanol, embedded in paraffin, and sectioned (4–6 μm thick). Cell samples were fixed directly on slides for subsequent processing. Paraffin sections were deparaffinized three times with xylene (5 min each), rehydrated in a series of ethanol gradients (100% → 95% → 85% → 70%) for 5 min each, and rinsed with distilled water for 5 min. Some tissues required protease K digestion or microwave antigen retrieval (95 °C sodium citrate buffer, pH 6.0, for 10 min). Cell membranes were permeabilized on ice with a mixture of 0.1% Triton X-100 and 0.1% sodium citrate for 10 min. After washing with PBS, the TUNEL reaction mixture (containing TdT enzyme and FITC-dUTP) was added dropwise and incubated at 37 °C in the dark for 1 h. The sections were then washed three times with PBS (5 min each) to remove unbound reagents, and apoptosis signals were observed using a fluorescence microscope or confocal microscope (excitation wavelength 488 nm, emission wavelength 530 nm).

### 2.16 Immunofluorescence

Paraffin-embedded brain sections from neonatal HIE mice were deparaffinized in xylene and rehydrated through a graded ethanol series. Antigen retrieval was performed by heating sections in citrate buffer at 95 °C for 10 min. After cooling, sections were washed three times with PBS (10 min per wash) to reduce autofluorescence. Sections were blocked with 10% goat serum and incubated overnight at 4 °C with primary antibodies against IBA1 (1:1000, Abclonal, A19776), iNOS (1:200, Abclonal, A3774), and CD206 (1:200, Abclonal, A21014). After PBS washes, sections were incubated with corresponding fluorescent secondary antibodies (1; 1000, Yeasen Biotechnology, Shanghai, China) in the dark at room temperature. Nuclei were counterstained with DAPI (Solarbio, Beijing, China) for 10 min and, after final washes, sections were mounted with anti-fade mounting medium. Images were acquired using a fluorescence microscope (OLYMPUS CKX53).

### 2.17 Statistical analysis

All experimental data were analyzed with strict adherence to randomization and blinding protocols to ensure unbiased results. Animals were allocated to experimental groups using stratified block randomization based on baseline weight and experimental endpoints, with sample processing order randomized across groups to eliminate batch effects. Investigators remained blinded to group assignments during data collection and analysis. All data were analyzed using GraphPad Prism 10 (GraphPad Software Inc., CA, United States) and are presented as mean ± SEM. All experiments were independently repeated at least three times. Post-hoc power analysis (G*Power, one-way ANOVA, α = 0.05) achieved power >0.999, indicating that the sample size is adequate for detecting the observed effects. For comparisons among three or more groups, one-way analysis of variance (ANOVA) followed by Tukey’s *post hoc* test was used when data met parametric assumptions; otherwise, the Kruskal–Wallis test followed by Dunn’s *post hoc* test was applied. Data normality was evaluated using the Shapiro–Wilk test. Statistical significance was set at *p* < 0.05.

## 3 Results

### 3.1 Effects of Dp3G on BV2 cell viability

To determine the effects of Dp3G on BV2 cell viability, cells were treated with various concentrations of Dp3G (20, 50, 80, 120, 180, and 250 μM) for 24 h. Results showed that Dp3G at concentrations ranging from 20 to 80 μM did not significantly affect BV2 cell viability compared with the control group (*p* > 0.05) (Figure 1B). Therefore, these non-cytotoxic concentrations were selected for subsequent experiments.

To establish an *in vitro* model of hypoxic-ischemic brain injury, BV2 cells were subjected to OGD for 4 h. The optimal therapeutic concentration of Dp3G was screened by treating OGD-injured BV2 cells with 20, 50, and 80 μM Dp3G. The CCK-8 assay revealed that OGD exposure significantly reduced BV2 cell viability (*p* < 0.001), whereas Dp3G treatment significantly restored cell viability (*p* < 0.05). Among the tested concentrations, 50 μM Dp3G produced the most pronounced protective effect (*p* < 0.001) (Figure 1C). Accordingly, 50 μM Dp3G was used for subsequent experiments.

### 3.2 Dp3G attenuates OGD-induced inflammation and oxidative stress by reprogramming BV2 polarization

**FIGURE 2:**
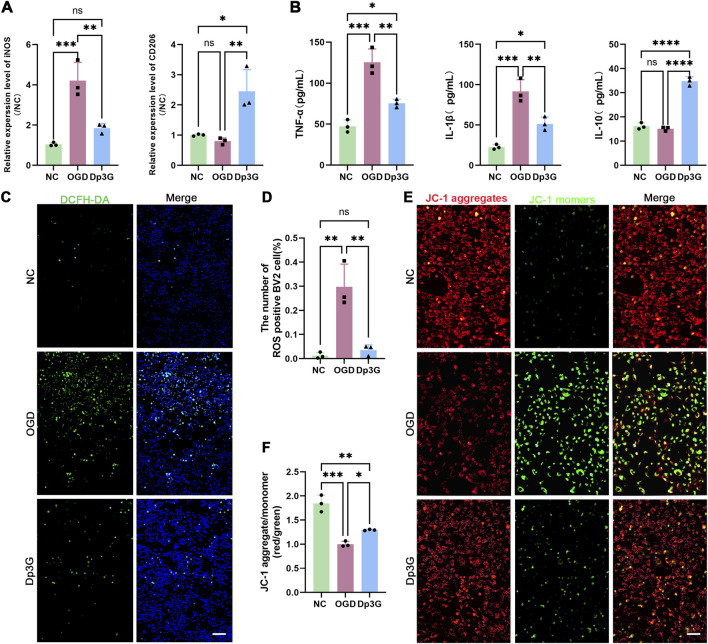
Dp3G reduces inflammatory and oxidative stress levels in BV2 cells by reprogramming their microglial polarization. **(A)** qRT-PCR analysis of iNOS and CD206 mRNA levels in the NC, OGD and Dp3G groups; **(B)** ELISA analysis of TNF-α, IL-1β and IL-10 levels in the NC, OGD and Dp3G groups; **(C,D)** DCFH-DA probe detection of ROS levels in BV2 cells from the NC, OGD and Dp3G groups, Green: DCFH-DA, Blue: Hoechst, scale bar: 50 μm; **(E,F)** JC-1 assay assessing mitochondrial membrane potential in HT22 cells of NC, OGD, and Dp3G groups (scale bar: 25 μm). Data represent the mean ± SEM from n = 3 per condition. Statistical significance was assessed using one-way ANOVA. *p < 0.05, **p < 0.01, ***p < 0.001.

To explore the mechanism by which Dp3G exerts protective effects in BV2 microglial cells, the expression of pro-inflammatory/anti-inflammatory polarization markers was first examined. qRT-PCR analysis showed that OGD exposure significantly upregulated inducible nitric oxide synthase (iNOS) expression (*p* < 0.001), whereas Dp3G treatment suppressed iNOS expression (*p* = 0.0043) and enhanced CD206 expression (*p* = 0.0072) (Figure 2A), indicating inhibition of pro-inflammatory polarization and promotion of anti-inflammatory polarization. Inflammatory and oxidative stress responses in BV2 cells were then assessed. ELISAanalysis revealed that Dp3G significantly suppressed OGD-induced increases in the expression of the pro-inflammatory cytokines TNF-α (*p* = 0.0029) and IL-1β (*p* = 0.0061) and enhanced the expression of the anti-inflammatory cytokine IL-10 (*p* < 0.001) (Figure 2B). Intracellular ROS levels were evaluated using the DCFH-DA fluorescent probe. Consistent with the inflammatory changes, Dp3G significantly attenuated OGD-induced ROS overproduction in BV2 cells (*p* = 0.003) (Figures 2C,D). These findings indicate that Dp3G inhibits OGD-induced inflammation and oxidative stress by reprogramming microglial polarization.

To investigate the effects of BV2 cells under different polarization states on neurons, conditioned medium was collected from differently treated BV2 groups and applied to HT22 neuronal cells for 24 h. Apoptosis was assessed by measuring mitochondrial membrane potential in HT22 cells using the JC-1 fluorescent probe. The results showed that HT22 cells in the Dp3G-treated group exhibited a significantly higher mitochondrial membrane potential than those in the OGD group (*p* = 0.0338), indicating reduced apoptosis (Figures 2E,F). These findings suggest that Dp3G mitigates neuronal damage by shifting microglial polarization from a pro-inflammatory toward an anti-inflammatory phenotype, thereby exerting anti-inflammatory and antioxidant effects.

In summary, these findings indicate that Dp3G not only directly regulates the transition of microglia from the pro-inflammatory pro-inflammatory phenotype to the anti-inflammatory anti-inflammatory phenotype but also attenuates neuronal damage through anti-inflammatory and antioxidant effects mediated by anti-inflammatory microglia. Collectively, these results highlight the role of Dp3G in alleviating neural injury by reprogramming the balance of microglial polarization.

### 3.3 RNA-seq analysis to elucidate the mechanism of Dp3G action

**FIGURE 3:**
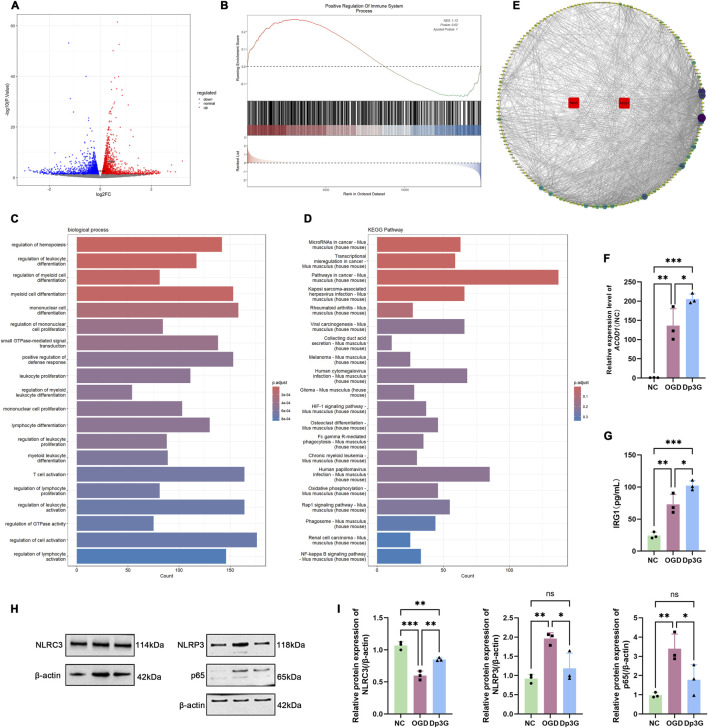
RNA-seq analysis to elucidate the mechanism of Dp3G action. **(A)** Volcano plot of differentially expressed genes (DEGs) between the OGD and Dp3G-treated groups. Upregulated and downregulated genes are highlighted in red and blue, respectively. Key upregulated genes of interest, *NLRC3* and *ACOD1*, are labeled; **(B)** GSEA enrichment plot for the “Positive Regulation Of Immune System Process” pathway derived from DEGs between the OGD and Dp3G-treated groups; **(C,D)** Bar graphs showing the top enriched **(C)** KEGG pathways and **(D)** GO biological process (BP) terms for DEGs between the Dp3G-treated and OGD groups in BV2 cells; **(E)** Protein–protein interaction (PPI) network constructed using the STRING database; **(F)** the mRNA expression level of *ACOD1* was determined by qRT-PCR; **(G)** IRG1 protein levels measured by ELISA; **(H,I)** the protein expression levels of NLRC3, NLRP3, and p65 assessed by Western Blot analysis. Data represent the mean ± SEM from n = 3 per condition. Statistical significance was assessed using one-way ANOVA. *p < 0.05, **p < 0.01, ***p < 0.001.

To investigate the molecular events triggered by Dp3G, gene expression profiles of BV2 cells in the Dp3G-treated group were compared with those of the OGD group. This analysis identified 2,251 upregulated and 2,089 downregulated differentially expressed genes (DEGs). The volcano plot highlighted the upregulation of key genes, including NLR family CARD domain containing 3 (NLRC3, also known as CLR16.2 or NOD3) (log_2_FC = 0.18, *p* < 0.001) and aconitate decarboxylase 1 (ACOD1, also known as CAD or IRG1) (log_2_FC = 0.33, *p* < 0.001) (Figure 3A).

To further explore the effects of Dp3G on microglia, Gene Set Enrichment Analysis (GSEA) was performed on a transcriptomic dataset comprising 21,677 genes. The results revealed significant enrichment in the positive regulation of the immune system process pathway (Figure 3B). Notably, KEGG pathway analysis indicated significant enrichment of the oxidative phosphorylation pathway in Dp3G-treated microglia under OGD conditions, consistent with reported metabolic reprogramming of microglia during inflammatory responses (Figure 3C). Similarly, Gene Ontology (GO) enrichment analysis demonstrated significant enrichment of biological processes related to immune cell regulation (Figure 3D). Subsequently, genes from the GSEA-enriched “Positive Regulation of Immune System Process” pathway were entered into the Search Tool for the Retrieval of Interacting Genes (STRING) database to construct a protein–protein interaction (PPI) network. This analysis revealed a highly interconnected network comprising 173 nodes and 1,484 edges (Figure 3E), suggesting extensive interactions within this gene set.

ACOD1 is a mitochondrial enzyme that catalyzes the production of itaconic acid. It was initially identified as a bacterial lipopolysaccharide (LPS)-induced gene involved in the innate immune response of mouse macrophages (Pålsson-Mcdermott and O’neill, 2025). The metabolic product of ACOD1, itaconate, has been shown to exert anti-inflammatory effects in various disease models as a key immunometabolic mediator (Liu et al., 2025; Peace and O’neill, 2022; Shi et al., 2022; Luo et al., 2024). The levels of ACOD1 mRNA and its encoded protein IRG1 were measured by qRT-PCR and ELISA. The results showed that Dp3G treatment significantly increased the expression levels of ACOD1 (*p* = 0.0428) and IRG1 (*p* = 0.0323) (Figures 3F,G). These findings suggest that Dp3G may enhance itaconic acid production by promoting oxidative phosphorylation in BV2 cells, thereby exerting anti-inflammatory effects. Given that multiple studies have reported a role for NLRC3 in suppressing activation of the NF-κB signaling pathway and inflammasome complex formation (Xu et al., 2023; Holley et al., 2018; Uchimura et al., 2018), the protein levels of NLRC3, p65, and NLRP3 in BV2 cells were assessed by Western blot. The results demonstrated that Dp3G treatment upregulated NLRC3 expression (*p* = 0.0034) and inhibited the OGD-induced increases in p65 (*p* = 0.0477) and NLRP3 (*p* = 0.0221) protein levels (Figures 3H,I). These data indicate that Dp3G likely alleviates inflammation in BV2 cells by activating NLRC3 and suppressing the NF-κB signaling pathway.

### 3.4 Molecular docking predicts potential targets of Dp3G

**FIGURE 4:**
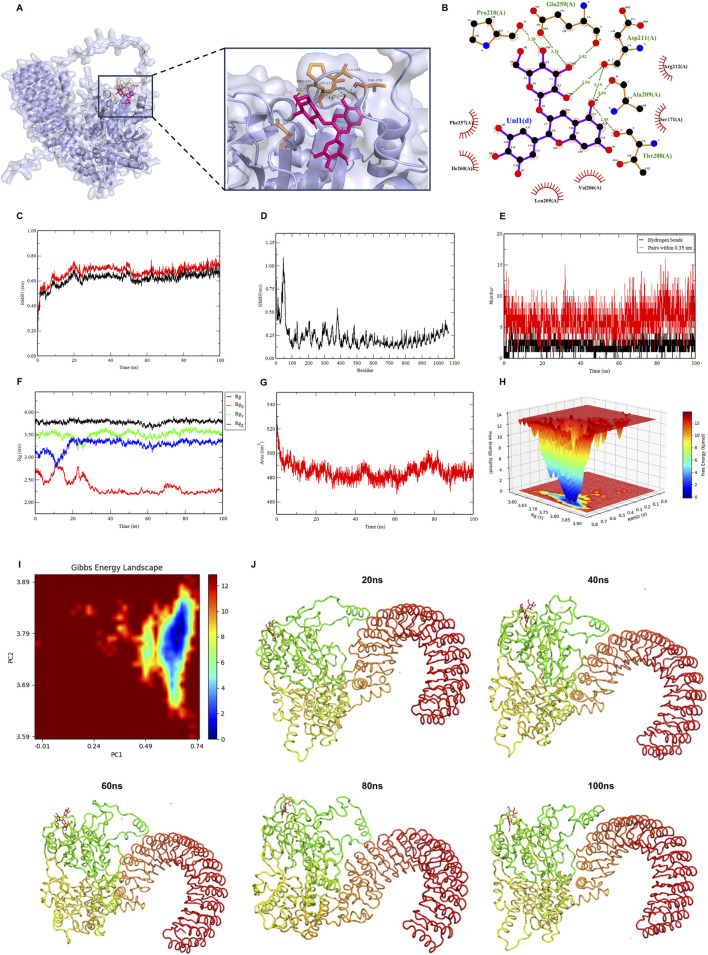
Molecular docking and molecular dynamics analyses predict potential targets of Dp3G. **(A)** Three-dimensional representation of the predicted binding pose of Dp3G within the NLRC3 binding pocket; **(B)** Two-dimensional schematic diagram illustrating key molecular interactions between Dp3G and NLRC3 residues, including hydrogen bonds and hydrophobic contacts; **(C)** Root mean square deviation (RMSD) of the Dp3G-NLRC3 complex over the 100 ns molecular dynamics (MD) simulation; **(D)** Root mean square fluctuation (RMSF) profile of the Dp3G-NLRC3 complex; **(E)** Number of intermolecular hydrogen bonds formed between Dp3G and NLRC3 during the 100 ns MD simulation; **(F)** Radius of gyration (Rg) of the Dp3G-NLRC3 complex throughout the MD simulation; **(G)** Solvent accessible surface area (SASA) of the Dp3G-NLRC3 complex during the MD simulation; **(H)** Free energy landscape of the Dp3G-NLRC3 complex shown as three-dimensional surface plot with RMSD and Rg as reaction coordinates; **(I)** Two-dimensional contour representation of the free energy landscape; **(J)** Representative conformational snapshots of the Dp3G-NLRC3 complex extracted at 20 ns intervals from the 100 ns MD simulation trajectory.

To examine the potential interaction between Dp3G and NLRC3, the molecular structure of Dp3G was obtained from the PubChem database. In parallel, the three-dimensional structure of NLRC3 predicted by AlphaFold was retrieved from the UniProt database. The reliability of this predicted structure was assessed using the SAVES server, which yielded an Overall Quality Factor of 91.1134. Molecular docking analysis identified a stable binding conformation of Dp3G within the NLRC3 receptor (Figures 4A,B). The calculated binding free energy of −7.20 kcal/mol indicates that Dp3G may function as a ligand for NLRC3.

To further characterize the conformational stability and local dynamics of the NLRC3-Dp3G complex, we performed a 100 ns molecular dynamics (MD) simulation. The MD trajectory indicated that the system reached equilibrium after approximately 70 ns, with the binding pocket retaining structural integrity, as evidenced by minimal fluctuations in the root mean square deviation (RMSD) (Figure 4C). The root mean square fluctuation (RMSF) profile showed that local flexibility was largely confined to the ligand-binding cavity, with minimal fluctuations elsewhere, supporting a stable binding mode (Figure 4D). Dynamic formation and dissociation of hydrogen bonds were observed throughout the simulation, with the number of hydrogen bonds ranging from 0 to 16 (Figure 4E). Analysis of the radius of gyration (Rg) indicated that the system maintained a compact conformation following initial equilibration, without significant structural expansion or unfolding (Figure 4F). Structural equilibration was further corroborated by a stable solvent-accessible surface area (SASA), indicating a compact and stable protein conformation throughout the simulation (Figure 4G). Free energy landscape analysis identified a global energy minimum at an RMSD of approximately 0.70 Å and an Rg of approximately 3.80 Å, representing the most thermodynamically stable conformational state of the complex (Figures 4H,I). Conformational cluster analysis of the trajectory demonstrated that the ligand adopted a stable binding pose, with its motion restricted to a narrow conformational space following complex formation (Figure 4J).

These computational results support a stable binding mode between Dp3G and NLRC3, providing a structural basis for its potential involvement in downstream signaling pathway activation.

### 3.5 Neuroprotective effects of Dp3G *in vivo*

To evaluate the therapeutic potential of Dp3G *in vivo,* a neonatal HIE model was established in postnatal day 7 mice by ligation of the right common carotid artery followed by 50 min of hypoxia. Model mice were randomly assigned to three groups: sham-operated (Sham), PBS-injected (HIE), and Dp3G-injected (Dp3G). Dp3G (20 mg/kg) was administered for three consecutive days after model induction. Mice were then anesthetized with isoflurane and euthanized for subsequent brain tissue analysis.

**FIGURE 5:**
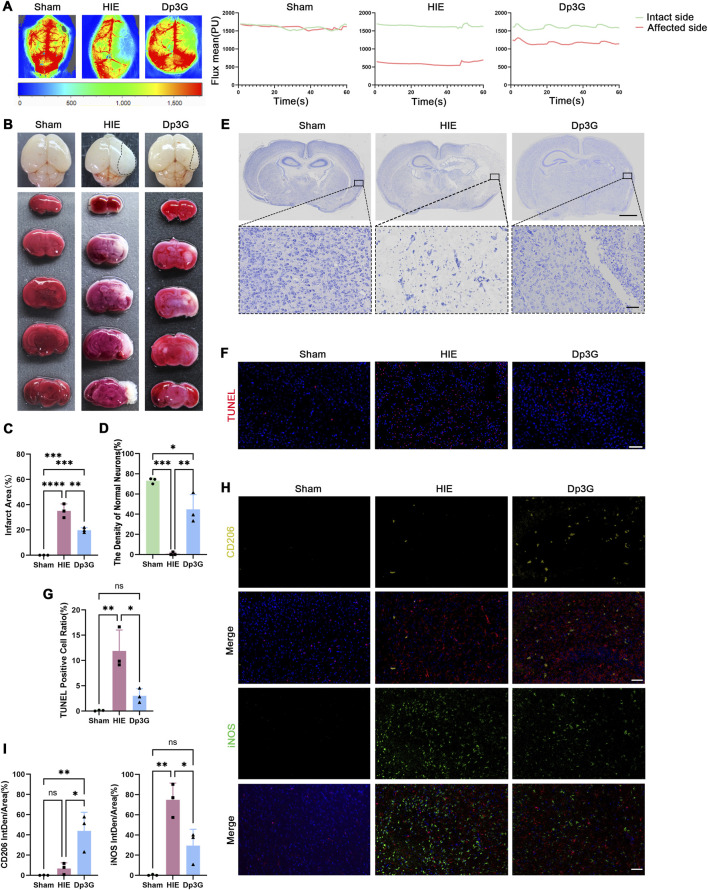
Neuroprotective effects of Dp3G *in vivo*. **(A)** Laser Doppler imaging of cerebral blood flow (rCBF) in Sham, HIE, and Dp3G mice; **(B**,**C)** Representative gross brain images and TTC-stained sections from Sham, HIE, and Dp3G mice, with dashed lines indicating edematous regions; **(D**,**E)** Representative Nissl-stained brain sections from Sham, HIE, and Dp3G groups (scale bar: 1 mm, 50 μm); **(F**,**G)** Representative TUNEL staining images and quantitative analysis of apoptotic cells in brain tissue from Sham, HIE, and Dp3G groups (red: TUNEL; blue: DAPI, scale bar: 75 μm); **(H,I)** Representative images and quantitative analysis of CD206 and iNOS immunofluorescence in brain tissue from Sham, HIE, and Dp3G groups (yellow: CD206, green: iNOS, red: IBA1, blue: DAPI, scale bar: 60 μm). Data represent the mean ± SEM from n = 3 per condition. Statistical significance was assessed using one-way ANOVA. *p < 0.05, **p < 0.01, ***p < 0.001.

Laser Doppler perfusion imaging revealed a near-complete loss of regional cerebral blood flow (rCBF) in the ipsilateral hemisphere of HIE mice, with the mean rCBF reduced by approximately 1040 perfusion units (PU) relative to the contralateral hemisphere. Dp3G treatment significantly improved cerebral perfusion, reducing the interhemispheric rCBF difference to 440 PU (Figure 5A; Supplementary Figure S1). TTC staining, which differentiates infarcted (white) from viable (red) tissue based on mitochondrial dehydrogenase activity, demonstrated extensive cerebral infarction and liquefactive necrosis in the HIE group, whereas Dp3G treatment markedly reduced infarct volume and cerebral edema (*p* = 0.0033) (Figures 5B,C). Together, these findings indicate that Dp3G attenuates ischemic brain injury and facilitates vascular recovery and the restoration of cerebral blood flow.

Nissl staining further demonstrated neuronal injury in the HIE group, characterized by pale cytoplasmic staining, dissolution of Nissl bodies, nuclear pyknosis, and indistinct cellular boundaries. In contrast, Dp3G treatment preserved neuronal morphology, with uniformly distributed dark blue Nissl bodies, clearly defined nuclear membranes, and prominent nucleoli, accompanied by a significant increase in the number of morphologically normal neurons (*p* = 0.002) (Figures 5D,E). TUNEL fluorescence staining further confirmed a significant reduction in apoptotic cells in the Dp3G-treated group compared with the HIE group (*p* = 0.0121) (Figures 5F,G), indicating robust anti-apoptotic and neuroprotective effects of Dp3G.

To determine whether the neuroprotective effects of Dp3G *in vivo* are mediated through reprogramming of microglial polarization, immunofluorescence staining was performed on brain tissue sections to assess microglial activation states. The results showed a marked increase in IBA1 (red) signal intensity in the HIE group, indicating extensive microglial activation (Figure 5H). Moreover, microglia exhibited a pronounced pro-inflammatory-skewed phenotype, characterized by elevated iNOS and reduced CD206 fluorescence intensity. In contrast, Dp3G treatment significantly suppressed iNOS expression (*p* = 0.0142) while enhancing CD206 signal intensity (*p* = 0.0151) (Figures 5H,I), indicating a shift from a pro-inflammatory pro-inflammatory phenotype toward an anti-inflammatory anti-inflammatory phenotype.

In summary, these *in vivo* findings demonstrate that Dp3G exerts significant neuroprotective effects in HIE by promoting anti-inflammatory anti-inflammatory polarization of microglia, thereby improving cerebral hemodynamics, reducing infarct volume, and attenuating neuronal apoptosis. These results highlight the potential of Dp3G as a therapeutic agent for hypoxic-ischemic brain injury.

## 4 Discussion

**FIGURE 6:**
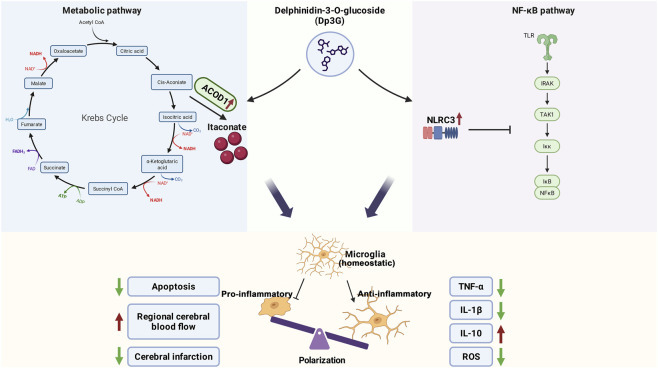
Proposed mechanism by which Dp3G mitigates hypoxic-ischemic brain injury. Dp3G regulates microglial polarization from the pro-inflammatory pro-inflammatory phenotype toward the anti-inflammatory anti-inflammatory phenotype through two complementary mechanisms: metabolic reprogramming and inhibition of the NF-κB signaling pathway. Metabolic reprogramming pathway (left): Dp3G enhances oxidative phosphorylation in microglia and promotes the tricarboxylic acid (TCA) cycle activity. Importantly, it upregulates the expression of ACOD1, leading to increased production of its metabolite itaconate, an anti-inflammatory mediator that contributes to suppression of inflammatory response. NF-κB pathway inhibition (right): Dp3G promotes NLRC3 expression, which in turn inhibits activation of the NF-κB signaling pathway. Integrated protective effects (bottom): The combined actions of these pathways drive microglial polarization toward the anti-inflammatory phenotype, resulting in reduced production of pro-inflammatory cytokines (TNF-α, IL-1β), decreased ROS levels, and increased secretion of the anti-inflammatory cytokine IL-10. These changes ultimately reduce neuronal apoptosis, limit cerebral infarct volume, and promote restoration of cerebral blood flow, thereby achieving neuroprotection and tissue repair.

This study systematically examined the neuroprotective role and underlying molecular mechanisms of the natural anthocyanin monomer Dp3G in hypoxic-ischemic brain injury using integrated *in vitro* and *in vivo* models. We demonstrated that Dp3G effectively reprograms microglial polarization, shifting microglia from a pro-inflammatory pro-inflammatory phenotype toward an anti-inflammatory anti-inflammatory phenotype. Transcriptomic analysis indicated that this functional transition is likely mediated through dual mechanisms: promoting metabolic reprogramming and increasing production of the anti-inflammatory metabolite itaconate, while concurrently upregulating NLRC3 to suppress the NF-κB pathway. Collectively, these actions rebalance microglial polarization, attenuate neuroinflammation and oxidative stress, and lead to marked reductions in pathological brain injury, restoration of cerebral blood flow, and overall neuroprotection (Figure 6).

Microglia-mediated neuroinflammation is a central driver of the secondary injury cascade in HIE. During the early phase of hypoxia-ischemia, microglia are rapidly activated and polarized toward the pro-inflammatory phenotype, releasing pro-inflammatory cytokines and ROS that exacerbate blood–brain barrier disruption and neuronal death (Chen et al., 2022; Zen et al., 2025; Bremer et al., 2025). Accordingly, shifting microglia from the detrimental pro-inflammatory state toward a protective anti-inflammatory phenotype has emerged as a promising therapeutic strategy. Our study demonstrates that Dp3G effectively promotes this polarization rebalancing. This finding aligns with previously reported anti-inflammatory properties of Dp3G in models such as atherosclerosis (Sun et al., 2022; Skemiene et al., 2015; Jin et al., 2013); however, we specifically position its role within central nervous system immune regulation and reveal its neuroprotective effect. As shown in *Results 2.2*, Dp3G promotes polarization of OGD-treated BV2 cells from pro-inflammatory to anti-inflammatory phenotypes, reducing inflammatory and oxidative stress levels. Moreover, conditioned medium from Dp3G-treated OGD-damaged microglia significantly reduced HT22 neuronal apoptosis compared with medium from untreated OGD microglia. This indicates that Dp3G exerts neuroprotective effects indirectly by reprogramming microglial polarization. Following Dp3G treatment, microglia reduce the release of inflammatory mediators such as TNF-α and IL-1β while increasing the secretion of neurotrophic or anti-inflammatory factors, thereby creating a more favorable microenvironment for neuronal survival. This provides a novel perspective on how natural products protect neurons by modulating neuroimmune crosstalk.

This study identified a novel mechanism underlying the effects of Dp3G through RNA-seq analysis. As shown in *Results 2.3*, both GSEA and KEGG analyses indicated that upregulation of oxidative phosphorylation and immune system regulation pathways represents key events following Dp3G treatment. In recent years, immunometabolism, the close link between immune cell metabolic states and functional phenotypes, has gained increasing attention: pro-inflammatory pro-inflammatory microglia primarily rely on glycolysis, whereas anti-inflammatory anti-inflammatory phenotypes depend more on oxidative phosphorylation (Sabogal-Guáqueta et al., 2023; Zhao et al., 2025; Jung et al., 2025; Cheng et al., 2025; Liu et al., 2025). Our findings suggest that Dp3G may promote a metabolic shift in microglia from glycolysis toward oxidative phosphorylation, thereby providing an energetic foundation for anti-inflammatory polarization. Meanwhile, restoration of itaconate levels, a key metabolite linking cellular metabolism and anti-inflammatory responses (Pålsson-Mcdermott and O’neill, 2025; Peace and O’neill, 2022; Shi et al., 2022; Luo et al., 2024), further supports the occurrence of metabolic reprogramming.

At the signaling level, this study links the anti-inflammatory effects of Dp3G to NLRC3-mediated suppression of the NF-κB pathway. NLRC3, a member of the NOD-like receptor family with a well-established role in negative regulation of inflammation, has been reported to inhibit NF-κB activation (Xu et al., 2023; Holley et al., 2018; Uchimura et al., 2018). Western blot analysis confirmed that Dp3G upregulates NLRC3 expression while concurrently downregulating p65 and NLRP3 levels. Importantly, molecular docking and MD simulations predicted a direct binding interaction between Dp3G and NLRC3 with high confidence (*Results 2.4*). Based on these findings, we propose a plausible mechanistic model in which Dp3G enhances inhibition of the NF-κB pathway through direct interaction with NLRC3, thereby attenuating the feed-forward cycle of neuroinflammation. This provides a new perspective on how natural products may selectively modulate inflammatory signaling pathways.

In our *in vivo* experiments, we confirmed the therapeutic efficacy of Dp3G at the systemic level. Despite the presence of the BBB, Dp3G can cross this barrier and exert therapeutic effects in central nervous system disease models, likely mediated by surface transporters on BBB endothelial cells (Dreiseitel et al., 2009). As shown in *Results 2.5*, Dp3G treatment significantly reduced cerebral infarct volume and edema, promoted restoration of cerebral blood flow, and markedly decreased neuronal apoptosis. Importantly, immunofluorescence analysis of brain tissue demonstrated that Dp3G effectively suppressed microglial pro-inflammatory polarization while promoting a shift toward the anti-inflammatory phenotype *in vivo*. These findings corroborate our *in vitro* results, establishing a coherent chain of evidence from cellular to animal levels and from phenotypic observation to mechanistic insight. Together, this identifies reprogramming of microglial phenotypic polarization as the central mechanism underlying the neuroprotective effects of Dp3G.

Nevertheless, this study has some limitations. First, although bioinformatic analysis and molecular docking predicted potential targets, specific binding of Dp3G to these targets and precise regulation of downstream signaling pathways require further validation through biochemical experiments (e.g., surface plasmon resonance, Western blot) and genetic knockout models (e.g., ACOD1 and NLRC3). Second, the pharmacokinetic profile of Dp3G *in vivo*, particularly BBB permeability, is crucial for evaluating its drug-like properties and represents an important direction for future research. Furthermore, long-term effects on neurobehavioral outcomes require verification in studies with extended observation periods. Finally, investigation of direct interactions between Dp3G and other brain cells, such as astrocytes and neurons, would provide a more comprehensive understanding of its protective mechanisms. Future studies could investigate potential synergistic effects between Dp3G and other well-established neuroprotective pathways. For instance, given the promising results of Nrf2 activation in promoting functional recovery after stroke (Hao et al., 2026), it would be intriguing to explore whether a combined regimen of Dp3G and Nrf2 agonists could yield augmented therapeutic benefits, potentially targeting microglia and neural stem cells simultaneously.

In conclusion, the findings of this study demonstrate meaningful translational potential. We not only propose a novel mechanism by which the natural active compound Dp3G counteracts HIE through reprogramming microglial polarization but also provide solid preclinical evidence supporting its potential as a neuroprotective agent. Future research focused on precise identification of molecular targets and subsequent preclinical development may offer new therapeutic strategies for neonatal HIE, a condition for which treatment options remain limited. Moreover, these findings may also inform therapeutic approaches for ischemic stroke across different age groups, given the shared core pathophysiology of microglia-mediated neuroinflammation.

## 5 Conclusion

This study identifies the natural compound Dp3G as a promising neuroprotective candidate for HIE treatment. We demonstrate, for the first time, that its protective effects are likely mediated by a dual mechanism involving microglial metabolic reprogramming and NLRC3-dependent inhibition of the NF-κB pathway. This coordinated action promotes a shift in microglial polarization from the pro-inflammatory pro-inflammatory phenotype to the anti-inflammatory anti-inflammatory phenotype, thereby conferring neuroprotection. Collectively, these findings advance the understanding of anthocyanin-mediated neuroprotection and provide a robust experimental and conceptual framework for the development of Dp3G-based therapeutic strategies targeting neuroimmune interactions in hypoxic-ischemic brain injury.

## Data Availability

The datasets presented in this study can be found in online repositories. The names of the repository/repositories and accession number(s) can be found below: https://ngdc.cncb.ac.cn/gsa, CRA033857.
