# Supplementary material for: Delphinidin-3-O-glucoside attenuates neonatal hypoxic-ischemic encephalopathy in a neonatal mouse model by reprogramming microglial polarization
Source: Front Pharmacol. 2026 May 4;17:1797078. doi: 10.3389/fphar.2026.1797078 (PMC13180932; doi:10.3389/fphar.2026.1797078)
Supplement: Supplementary file 1 [file DataSheet1.pdf]

Supplementary Material

1 Supplementary Figures and Tables

1.1 Supplementary Figures

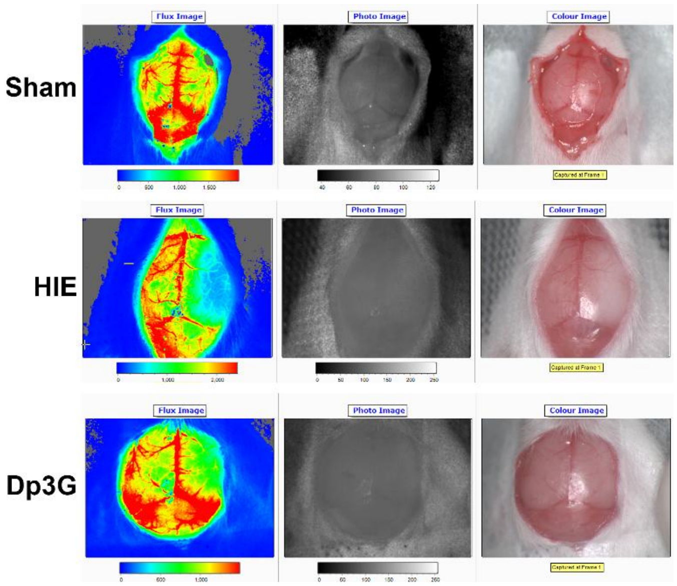

Supplementary Figure 1. Flux image, photo image and color image of rCBF in Sham, HIE and Dp3G mice.

1.2 Supplementary Tables

Gene	Forward primer (5'→3')	Reverse primer (5'→3')
iNOS	GGAGTGACGGCAAACATGACT	TCGATGCACAACTGGGTGAAC
CD206	GTTCACCTGGAGTGATGGTTCTC	AGGACATGCCAGGGTCACCTTT
ACOD1	CCAGCAGGATGTGGCCTTTA	GATGTGGTCAGCAGGGAACA
β-actin	CATTGCTGACAGGATGCAGAAGG	TGCTGGAAGGTGGACAGTGAGG

Supplementary Table 2. Primer sequences used for qRT-PCR.
